# Relationship between intake and plasma concentrations of vitamin B12 and folate in 873 adults with a physically active lifestyle: a cross‐sectional study

**DOI:** 10.1111/jhn.12814

**Published:** 2020-09-21

**Authors:** A. M. Baart, M. G. J. Balvers, J. H. M. de Vries, D. S. M. ten Haaf, M. T. E. Hopman, J. M. T. Klein Gunnewiek

**Affiliations:** ^1^ Division of Human Nutrition and Health Wageningen University & Research Wageningen The Netherlands; ^2^ Clinical Chemistry and Haematology Laboratory Gelderse Vallei Hospital Ede The Netherlands; ^3^ Department of Physiology Radboud University Medical Center Nijmegen The Netherlands

**Keywords:** vitamin B12, folate, intake, plasma concentrations, physical activity

## Abstract

**Background:**

Vitamin B12 and folate function as co‐factors in pathways used during physical activity. Physical activity may therefore increase vitamin requirements, leading to a risk of deficient plasma concentrations. We aimed to investigate the relationship between intake and plasma concentrations of vitamin B12 and folate in physically active adults, as well as identify other determinants of vitamin B12 and folate plasma concentrations.

**Methods:**

The study population consisted of 873 adults (528 men and 345 women), aged 19–78 years, who participated in a 4‐day walking event. The relationship between intake and plasma concentrations of vitamin B12 and folate was assessed using correlation and linear regression analyses. In addition, potential other determinants (sex, age, body mass index, energy intake and physical activity) of vitamin plasma concentrations were investigated.

**Results:**

Significant positive correlations were observed between intake and plasma concentrations of vitamin B12 [Pearson’s correlation coefficient = 0.15; 95% confidence interval (CI) = 0.08–0.21] and folate (Pearson’s correlation coefficient = 0.18; 95% CI = 0.12–0.25). In addition to vitamin intake, sex, age and energy intake were also determinants of both vitamin B12 and folate plasma concentrations in multivariable regression models.

**Conclusions:**

The results suggest a positive association between intake and plasma concentrations for both vitamin B12 and folate in physically active people. By contrast to our hypothesis, physical activity was not a determinant of vitamin B12 and folate plasma concentrations. However, sex, age and energy intake were found to be determinants. Thus, when studying the relationship between intake and plasma concentrations of vitamin B12 or folate, these factors should be taken into account.

## Introduction

B‐vitamins, such as vitamin B12 and folate, are essential for DNA synthesis and function as co‐factors in various metabolic processes. Because of their role in DNA synthesis, B‐vitamins are required for the synthesis of new cells, particularly red blood cells, as well as in the repair of damaged tissue. A deficiency in these micronutrients will ultimately impair cellular proliferation and function, leading to fatigue and pathophysiological conditions such as anaemia and neurological disorders ^(^ ^1^, ^2^, ^3^ ^)^.

A positive association between B‐vitamin intake and plasma concentrations has been reported in various studies ^(^ ^4^, ^5^ ^)^. However, vitamin plasma concentrations depend not only on vitamin intake, but also on vitamin uptake and vitamin metabolism. Because of their role in important pathways used during and after physical activity, it has previously been hypothesised that physical activity leads to increased requirements for B‐vitamins, and/or that an increased intake of B‐vitamins leads to improved recovery and performance ^(^ ^3^ ^)^. Correspondingly, physical activity might increase the risk of deficient plasma concentrations of B‐vitamins. However, evidence for these hypotheses is lacking.

Although B‐vitamin intake and/or plasma concentrations have been extensively investigated in various physically active populations, in particular among (professional) athletes and military personnel ^(^ ^3^ ^)^, only a few studies have included data on both vitamin intake and vitamin plasma concentrations from the same subjects ^(^ ^6^, ^7^ ^)^, which are necessary considerations for correctly interpreting vitamin status. In these studies, no deficient plasma concentrations of vitamin B12 and folate were observed among physically active people, nor were any differences in vitamin plasma concentrations observed between subjects with relatively low versus relatively high physical activity. However, the study populations in these studies were small (*n* = 58 ^(^ ^7^ ^)^ and *n* = 76 ^(^ ^6^ ^)^ subjects) and consisted of a specific group of athletes ^(^ ^6^ ^)^ or were limited to only women ^(^ ^7^ ^)^. If physical exercise leads to a higher turnover of B‐vitamins, this should become apparent when the relationship between intake and plasma concentrations is evaluated across a range of physical activity levels within the same study population. To our knowledge, the relationship between intake and plasma concentrations of vitamin B12 and folate has never been investigated in the general population at the same time as taking into account the level of physical activity.

In the present study, we aimed to investigate the relationship between intake and plasma concentrations of vitamin B12 and folate, in a large study population. The population consisted of Dutch adults with a physically active lifestyle, in whom the levels of physical activity covered a broad range. In addition, we aimed to identify other determinants of vitamin B12 and folate plasma concentrations, including physical activity. We hypothesised that physical activity leads to a higher turnover of vitamin B12 and folate and is an important determinant of plasma concentrations of these vitamins.

## Materials and methods

### Study population

The study population for the present study consisted of participants of the Nijmegen Four Days Marches 2015, an annual walking event in the Netherlands. Participants were recruited via newsletters and internet advertisements. In total, 1038 participants volunteered to participate in blood tests, as well as complete the questionnaire from the Nijmegen Exercise Study ^(^ ^8^ ^)^. This is an ongoing online questionnaire ^(^ ^9^ ^)^, which includes a food frequency questionnaire (FFQ) and questions about supplement intake, demographic characteristics, anthropometric measures, lifestyle factors, physical activity and health status. Because we aimed to have a study population with a physically active lifestyle, 23 subjects with a physical activity level below 500 metabolic equivalent of task (MET) minutes week^–1^ were excluded. This threshold level was based on international physical activity recommendations of 500–1000 MET minutes week^–1^^(^ ^10^ ^)^. In addition, 135 subjects who used vitamin B12, folate or multivitamin supplements were excluded. Finally, six subjects appeared to have extremely high plasma concentrations of vitamin B12 (>1000 pmol L^−1^) and one subject had an extremely high plasma concentration of folate (>200 nmol L^−1^). These values were considered abnormal; subjects with these extreme values were excluded because the cause could not be identified and these values may erroneously influence the statistical analyses. Finally, in total, 873 Nijmegen Four Days Marches participants (528 men and 345 women; aged 19–87 years) were enrolled in the present study.

The study was conducted in accordance with the Declaration of Helsinki guidelines for medical research, and all procedures involving research study participants were approved by the Medical Ethical Committee of the Radboud University Medical Center (file number: 2011/193; approval number: NL36743.091.11). Written informed consent was obtained from all subjects. Data were anonymously obtained and analysed.

### Assessment of vitamin B12 and folate plasma concentrations

Venous blood samples were collected 1 or 2 days before the start of the first day of the Four Days Marches (21 July 2015), in the morning or in the afternoon. The plasma of these samples was used on the same day to determine vitamin B12 and folate concentrations. Analysis was performed at the Clinical Chemistry and Haematology Laboratory of the Gelderse Vallei Hospital. Vitamin B12 concentrations were determined on the Advia Centaur XP platform using the Acridinium Ester chemiluminescence vitamin B12 assay (Siemens, Erlangen, Germany). Folate concentrations were determined on the Vista Dimension 1500 platform using the LOCI Folate method (Siemens). Both tests were performed in accordance with the manufacturer’s recommendations. Calibrators were provided as part of the assay (Siemens), and external quality control (QC) samples (Immunoassay Plus control; Bio‐Rad Laboratories, Veenendaal, the Netherlands) were analysed daily at two levels to monitor the analytical performance. Both assays performed within the acceptance criteria that were set by the manufacturer. The average imprecision of the QC analysis was 8.4% for vitamin B12 and 10.0% for folate (year‐averages for 2015). In addition, the laboratory participated in an external quality assessment scheme (Dutch Foundation for Quality Assessment in Medical Laboratories; SKML), which offers six surveys per year, each containing two samples. The imprecision and accuracy were 3.8% and 98.1%, respectively, for vitamin B12; and 5.3% and 102.1%, respectively, for folate (year‐averages for 2015). The methods were previously validated using external QC materials (Bio‐Rad) in accordance with the EP‐9 and EP‐10 protocols from the Clinical Laboratory Standards Institute (CLSI).

### Assessment of potential determinants of vitamin B12 and folate plasma concentrations

Data on sex, age, self‐reported body height and weight, physical activity, energy intake and vitamin B12 and folate intake were obtained from the Nijmegen Exercise Study ^(^ ^9^ ^)^.

Physical activity in the past month was assessed with a short questionnaire to assess health‐enhancing physical activity (SQUASH). The SQUASH has been shown to be substantially correlated with physical activity measured by accelerometry (correlation coefficient = 0.45) ^(^ ^11^ ^)^. Using the SQUASH, the average number of minutes per week of walking, cycling, carrying out household chores, gardening, doing odd jobs and sports activities was assessed. Based on Ainsworth’s compendium of physical activities ^(^ ^12^ ^)^, metabolic equivalent of task (MET) values were assigned to the specific physical activities. Subsequently, MET minutes week^–1^ were calculated by multiplying the minutes of physical activities with the accompanying MET values.

Dietary intake in the past month was assessed with a comprehensive FFQ that was validated for energy intake, macronutrients, dietary fibre and vitamins ^(^ ^13^, ^14^ ^)^. Pearson’s correlation coefficients (crude and adjusted for both energy intake and within‐person variation, respectively) for 24‐h dietary recalls were 0.43 and 0.72 for vitamin B12, and 0.53 and 0.87 for folate equivalents ^(^ ^14^ ^)^. With the FFQ the frequency of consumption of 180 food items during the previous month was assessed. Portion sizes were estimated using natural portions (e.g. one slice of bread) and commonly used household measures. Data obtained from the FFQ was converted into average daily energy and nutrient intake using data from the Dutch food composition database of 2010 ^(^ ^15^ ^)^. The intake of folate equivalents was calculated as: µg naturally present folate + µg synthetic folic acid from fortified foods × 1.7 (+ µg folic acid from food supplements × 2.0)^(^ ^16^ ^)^. Note that the part of the formula in parenthesis is not applicable because we excluded subjects who used folate supplements. Possible under‐reporting of dietary intake was evaluated using the Goldberg cut‐off method ^(^ ^17^, ^18^ ^)^. The reported energy intake (EI) divided by the estimated basal metabolic rate (BMR) according to Schofield’s formula ^(^ ^19^ ^)^, the EI/BMR ratio, was also calculated. This ratio was then compared with a cut‐off limit of 1.55 on the group level, and with a cut‐off limit of 0.87 on an individual level. The EI/BMR ratio had a mean (SD) of 1.44 (0.44), suggesting a mean underestimation of less than 10%, taking 1.55 as the cut‐off limit. A total of 52 subjects (6.0%) had an EI/BMR ratio below 0.87. Sensitivity analyses comparing the results with and without these 52 subjects showed similar results; thus, subjects who possibly under‐reported were not excluded.

### Statistical analysis

Descriptive analyses were performed first. Because of non‐normal distributions, median values and 25th to 75th percentiles of population characteristics, energy intake, intake of vitamin B12 and folate, and plasma concentrations of vitamin B12 and folate were calculated. Prevalence rates of nutrient intake below the estimated average requirement (EAR) in accordance with the recommendations of the Nordic Council (1.4 µg day^–1^ for vitamin B12 and 200 µg day^–1^ for folate equivalents) ^(^ ^20^ ^)^ were calculated using the cut‐point method ^(^ ^21^ ^)^. Prevalence rates of plasma concentrations below and above reference ranges were calculated in accordance with the reference ranges used in the Gelderse Vallei Hospital (150–600 pmol L^−1^ for vitamin B12 and 7–40 nmol L^−1^ for folate). These values are based on previous reports in the literature ^(^ ^22^ ^)^ and on results of laboratory tests in which vitamin B12 values were related to methylmalonic acid values.

The relationship between vitamin B12 and folate intake and their respective plasma concentrations was assessed using a Pearson correlation analysis and a linear regression analysis. In the regression analysis, variables also studied as potential determinants of vitamin B12 and folate plasma concentrations, in addition to vitamin B12 and folate intake, were: sex, age, body mass index (BMI), physical activity and energy intake. Univariable and multivariable regression models were fitted. In the multivariable models, potential interaction effects between the different determinants were also examined. Here, the variables age, BMI, physical activity and energy intake were first analysed categorised into quartiles. Full models containing all the main effects and all of the interaction effects were fitted. A backward stepwise selection procedure was then used to select significant (*P* < 0.05) determinants of vitamin B12 or folate plasma concentrations. When none of the interaction terms appeared to be significant, all determinants were analysed as continuous variables. New full models containing only all of the main effects were fitted and significant determinants were selected with backward selection. Because the residuals in the models were not normally distributed, we applied a base ‘*e*’ logarithmic transformation on the plasma concentrations of vitamin B12 and folate.

To investigate whether observed associations between determinants and vitamin plasma concentrations could be the result of a difference in vitamin intake, we compared median vitamin intake between men and women, as well as between the different quartiles of the investigated determinants. Differences between men and women were compared using a Mann–Whitney *U*‐test and differences between quartiles of other determinants were compared using a Kruskal–Wallis test, followed by Dunn’s post‐hoc tests with Bonferroni correction.

Finally, to obtain more insight into the relationship between vitamin intake and plasma concentrations, median vitamin plasma concentrations were calculated for the different quartiles of vitamin intake. These were also compared using a Kruskal–Wallis test, followed by Dunn’s post‐hoc tests with Bonferroni correction.

Statistical analyses were performed with SPSS software (Version 23, IBM, Armonk, NY, USA).

## Results

The study population characteristics are presented in Table 1. The median physical activity level of this physically active population was 7005 (25th to 75th percentile: 4215–9965) MET minutes week^–1^. The median vitamin B12 intake was 4.6 (25th to 75th percentile: 3.5–6.3) µg day^–1^ and the median folate equivalents intake was 296 (25th to 75th percentile: 239–373) µg day^–1^. The median plasma concentration of vitamin B12 was 272 (25th to 75th percentile: 221–327) pmol L^−1^ and the median plasma concentration of folate was 16.7 (25th to 75th percentile: 12.5–22.7) nmol L^−1^. The prevalence of inadequate vitamin intake was low (0.1% for vitamin B12 and 12.0% for folate equivalents) and more than 95% of the population had vitamin plasma concentrations within the reference range.

**Table 1:** Study population characteristics (*n* = 873)

Characteristic	Median or *n* ^†^	25th to 75th percentile or %^†^
Sex		
Men	528	60.5
Women	345	39.5
Age (years)	63	56–67
Height (m)	1.75	1.68–1.80
Weight (kg)	76.0	67.0–84.0
Body mass index (kg m^−2^)	24.7	22.9–26.9
Physical activity (MET minutes week^–1^)	7005	4215–9965
Energy intake (kcal)	2128	1766–2512
Vitamin intake per day		
Vitamin B12 (µg)	4.6	3.5–6.3
Folate equivalents (µg)	296	239–373
Inadequate intake related to EAR		
Vitamin B12 (<1.4 µg per day)	1	0.1
Folate equivalents (<200 µg per day)	105	12.0
Vitamin plasma concentrations		
Vitamin B12 (pmol L^−1^)	272	221–327
Folate (nmol L^−1^)	16.7	12.5–22.7
Vitamin plasma concentrations related to reference ranges		
Vitamin B12		
Below reference range (<150 pmol L^−1^)	14	1.6
Within reference range (150–600 pmol L^−1^)	851	97.5
Above reference range, (>600 pmol L^−1^)	8	0.9
Folate		
Below reference range (<7 nmol L^−1^)	14	1.6
Within reference range (7–40 nmol L^−1^)	833	95.4
Above reference range (>40 nmol L^−1^)	26	3.0

Abbreviations: BMI, body mass index; EAR, estimated average requirement; MET, metabolic equivalent of task.

^†^ Continuous variables are presented as medians and 25th to 75th percentiles; categorical variables are presented as numbers and percentages.

The correlation between intake and plasma concentrations was weak for both vitamin B12 and folate. Pearson’s correlation coefficient for intake and plasma concentrations of vitamin B12 was 0.15 [95% confidence interval (CI) = 0.08–0.21] (*P* < 0.001). For intake and plasma concentrations of folate, Pearson’s correlation coefficient was 0.18 (95% CI = 0.12–0.25) (*P* < 0.001).

Table 2 presents the results of the univariable and multivariable regression analyses with vitamin B12 plasma concentrations as the dependent variable. Univariably, age and vitamin B12 intake had a positive association with plasma concentrations of vitamin B12. In the multivariable model, age and vitamin B12 intake had a positive association, whereas male sex and energy intake were negatively associated with plasma concentrations of vitamin B12. BMI and physical activity were not significantly associated with plasma concentrations of vitamin B12 in the multivariable model. The explained variance (*r* ^2^) of the multivariable model was 0.050.

**Table 2:** Associations between investigated determinants and vitamin B12 plasma concentrations

Characteristic	Β (95% CI)
Univariable	
Sex (men)	−0.033 (−0.074 to 0.007)
Age (years)	0.002* (0.000 to 0.004)
BMI (kg m^−1^)	−0.003 (−0.009 to 0.003)
Physical activity (MET minutes week^–1^)	0.000 (−0.000 to 0.000)
Energy intake per day (kcal)	0.000 (−0.000 to 0.000)
Vitamin B12 intake per day (µg)	0.018* (0.011 to 0.026)
Multivariable^†^	
Intercept	5.422* (5.291 to 5.554)
Sex (men)	−0.063* (−0.106 to −0.020)
Age (years)	0.003* (0.001 to 0.005)
Energy intake per day (kcal)	−0.000* (−0.000 to −0.000)
Vitamin B12 intake per day (µg)	0.025* (0.017 to 0.034)

The beta represents the difference in the log *e* transformed predicted value of vitamin B12 plasma concentrations in pmol L^−1^ for 1 µg increase of vitamin B12 intake. Thus, a beta of 0.018 for vitamin B12 intake means that, for a 1 µg increase of vitamin B12 intake, the vitamin B12 plasma concentration increases by exp(0.018) = 1.018 nmol L^−1^, which corresponds to an increase of 1.8%.

Abbreviations: BMI body mass index; CI, confidence interval; MET metabolic equivalent of task.

* Significant determinant of vitamin B12 plasma concentrations (*P* < 0.05).

^†^ The final multivariable model after backward selection of significant determinants is presented.

The results of the univariable and multivariable regression analyses, with folate plasma concentrations as dependent variable, are presented in Table 3. Univariably, age and folate equivalents intake had a positive association with plasma concentrations of folate, whereas male sex and BMI had a negative association. In the multivariable model, age, energy intake and folate equivalents intake were positively associated, whereas male sex was negatively associated with plasma concentrations of folate. BMI and physical activity were not significantly associated with plasma concentrations of folate in the multivariable model. The *r* ^2^ of the multivariable model was 0.116.

**Table 3:** Associations between investigated determinants and folate plasma concentrations

Characteristic	Β (95% CI)
Univariable	
Sex (men)	−0.084* (−0.144 to −0.024)
Age (years)	0.007* (0.005 to 0.010)
BMI (kg m^−1^)	−0.014* (−0.023 to −0.005)
Physical activity (MET minutes week^–1^)	0.000 (−0.000 to 0.000)
Energy intake per day (kcal)	−0.000 (−0.000 to 0.000)
Folate equivalents intake per day (µg)	0.001* (0.001 to 0.001)
Multivariable^†^	
Intercept	2.293* (2.106 to 2.481)
Sex (men)	−0.120* (−0.182 to −0.058)
Age (years)	0.008* (0.005 to 0.010)
Energy intake per day (kcal)	0.000* (0.000 to −0.000)
Folate equivalents intake per day (µg)	0.001* (0.001 to 0.002)

The beta represents the difference in the log‐e transformed predicted value of folate plasma concentrations in nmol L^−1^ for 1 µg increase of folate equivalents intake. Thus, a beta of 0.001 for folate equivalents intake means that, for a 1 µg increase of folate equivalents intake, the folate plasma concentration increases by exp(0.001) = 1.001 nmol L^−1^, which corresponds to an increase of 0.1%.

Abbreviations: BMI, body mass index; CI, confidence interval; MET, metabolic equivalent of task.

* Significant determinant of folate plasma concentrations (*P* < 0.05).

^†^ The final multivariable model after backward selection of significant determinants is presented.

Table 4 presents the vitamin intake in men and women separately, and in different quartiles of the investigated determinants of vitamin plasma concentrations. Men had a higher intake of both vitamin B12 and folate equivalents compared to women, older subjects had a higher folate equivalents intake compared to younger subjects, and a higher energy intake was accompanied by a higher intake of both vitamin B12 and folate equivalents.

**Table 4:** Median daily intake of vitamin B12 and folate equivalents per sex and per quartile of investigated determinant of vitamin plasma concentrations

Quartile/Sex	Sex	Age	BMI	Physical activity	Energy intake
	*Vitamin B12 (µg)*	*Vitamin B12 (µg)*	*Vitamin B12 (µg)*	*Vitamin B12 (µg)*	*Vitamin B12 (µg)*
1 Men	4.9 (3.7–6.7)*	4.6 (3.5–6.1)	4.7 (3.4–6.2)	4.3 (3.4–5.9)	3.3 (2.6–4.6)* ^2,3,4^
2 Women	4.2 (3.3–5.6)*	4.6 (3.4–6.5)	4.6 (3.4–6.1)	4.6 (3.5–6.1)	4.5 (3.6–6.1)* ^1,4^
3		4.6 (3.5–6.1)	4.8 (3.6–6.7)	4.9 (3.8–6.7)	4.9 (4.0–6.2)* ^1,4^
4		4.7 (3.6–6.9)	4.5 (3.5–6.0)	4.5 (3.4–6.4)	6.1 (4.5–8.4)* ^1,2,3^
	*Folate equivalents (µg)*	*Folate equivalents (µg)*	*Folate equivalents (µg)*	*Folate equivalents (µg)*	*Folate equivalents (µg)*
1 Men	304 (244–388)*	278 (227–346)* ^4^	294 (251–387)	298 (228–361)	231 (185–281)* ^2,3,4^
2 Women	286 (234–357)*	296 (243–369)	307 (238–386)	294 (239–346)	278 (237–344)* ^1,3,4^
3		301 (238–385)	297 (242–362)	301 (241–391)	312 (260–371)* ^1,2,4^
4		307 (254–397)* ^1^	283 (231–363)	293 (245–378)	388 (314–463)* ^1,2,,3^

Values of vitamin intake are presented as the median (25th to 75th percentile).

Abbreviation: BMI, body mass index.

* Significant difference between men and women and between quartiles of investigated determinants (*P* < 0.05). The number after the asterisk (*) denotes the quartile from which the given quartile is significantly different.

Table 5 presents the vitamin plasma concentrations in different quartiles of vitamin intake. In general, a higher vitamin intake was accompanied by higher vitamin plasma concentrations.

**Table 5:** Median plasma concentrations of vitamin B12 and folate per quartile of vitamin intake

Quartile of vitamin intake	Vitamin intake per day	Vitamin plasma concentrations
	*Vitamin B12 (µg)*	*Vitamin B12 (pmol L^−1^)*
1	<3.5	260 (212–307)* ^3,4^
2	≥3.5 and <4.6	251 (215–312)* ^3,4^
3	≥4.6 and <6.3	281 (237–333)* ^1,2^
4	≥6.3	290 (235–350)* ^1,2^
	*Folate equivalents (µg)*	*Folate (nmol L^−1^)*
1	<239	14.7 (10.9–19.5)* ^3,4^
2	≥239 and <296	15.0 (12.5–21.2)* ^3,4^
3	≥296 and <373	17.8 (13.2–24.7)* ^1,2^
4	≥373	19.5 (14.6–25.0)* ^1,2^

Vitamin plasma concentrations are presented as the median (25th to 75th percentile).

* Significant difference between quartiles of vitamin intake (*P* < 0.05). The number after the asterisk (*) denotes the quartile from which the given quartile is significantly different.

## Discussion

The present study aimed to assess the relationship between intake and plasma concentrations of vitamin B12 and folate, as well as identify determinants of vitamin B12 and folate plasma concentrations, in a large population of adults with a physically active lifestyle. We observed a positive association between intake and plasma concentrations for both vitamin B12 and folate. By contrast to our hypothesis, physical activity was not a determinant of vitamin B12 and folate plasma concentrations. On the other hand, sex, age and energy intake were found to be determinants of both vitamin B12 and folate plasma concentrations in the multivariable regression models.

The observed positive association between vitamin B12 and folate equivalents intake and their respective plasma concentrations is in agreement with observations in previous studies ^(^ ^4^, ^5^ ^)^. However, it should be noted that these studies have been conducted in various study populations, and are not limited to physically active populations. We observed weak correlation coefficients for vitamin intake and plasma concentrations (0.15 for vitamin B12 and 0.18 for folate), which is also in agreement with the literature. For intake and plasma concentrations of vitamin B12, correlation coefficients ranging from 0.06 to 0.21 have been reported ^(^ ^23^, ^24^, ^25^ ^)^. In a review of 17 studies on the validity of intake and measurement of folate, correlation coefficients ranged from 0.05 to 0.54 ^(^ ^26^ ^)^. An explanation for the broad range in correlation coefficients for folate is that the correlations were considerably higher in studies where supplement use was taken into account. Also, stronger correlations with plasma folate concentrations have been reported for folic acid from fortified foods compared to naturally occurring food folate ^(^ ^27^ ^)^. This is likely a consequence of the higher bioavailability of synthetic folic acid than that of natural food folate ^(^ ^28^ ^)^. To adjust for these differences in bioavailability, it is recommended to express folate intake as dietary folate equivalents, which we did in the present study.

Male sex showed a significant negative association with both vitamin B12 and folate plasma concentrations in the multivariable models, whereas, in the univariable models, the negative association was only significant for folate plasma concentrations. Lower concentrations of vitamin B12 in men compared to women have been reported in certain studies ^(^ ^29^, ^30^, ^31^ ^)^, although not in all ^(^ ^32^ ^)^. Likewise, for folate concentrations, lower concentrations have been observed in men compared to women ^(^ ^30^, ^31^ ^)^, although not in all studies ^(^ ^29^, ^32^ ^)^. It is worth noting that most of these studies were conducted in elderly people. In a study among African Americans aged 21–94 years, lower vitamin B12 and folate concentrations in men compared to women were observed across different age groups, except for the 21–34‐year‐old age group ^(^ ^33^ ^)^. The negative association of male sex with vitamin plasma concentrations cannot be explained by differences in vitamin intake between men and women. Moreover, vitamin intake in men was even higher than in women. An explanation for these opposing observations could be that men have a larger body size and therefore a larger distribution volume, or that there is a difference in lean body mass between men and women ^(^ ^34^ ^)^. Another possible explanation for the observed association between sex and vitamin plasma concentrations could be hormonal effects. Variations in folate concentrations during different phases of the ovarian cycle have been reported ^(^ ^35^ ^)^. Also, lower concentrations of vitamin B12 in women using oral contraceptives compared to non‐users have been reported ^(^ ^36^, ^37^ ^)^. The mechanism by which oral contraceptives reduce vitamin B12 concentrations is unclear, although a decreasing effect on vitamin B12 binding capacity in serum has been suggested as an explanation ^(^ ^38^ ^)^. Although the results of these studies cannot explain the association between sex and vitamin plasma concentrations in the present study, these studies indicate that gonadal steroids might influence vitamin plasma concentrations.

We observed a significant positive association with age for both vitamin B12 and folate plasma concentrations in the univariable and in the multivariable models. This is in agreement with the study among African Americans ^(^ ^33^ ^)^, in which a general increase in vitamin B12 and folate concentrations was seen with older age. An explanation for the positive association between age and folate plasma concentrations could be a higher folate equivalents intake with increasing age. However, a higher vitamin B12 intake with increasing age was not observed, and so this cannot explain the positive association between age and vitamin B12 plasma concentrations.

In the multivariable models, energy intake showed a significant negative association with vitamin B12 plasma concentrations, and a significant positive association with folate plasma concentrations. However, in the univariable models, opposite associations were observed: energy intake showed a non‐significant positive association with vitamin B12 plasma concentrations and a significant negative association with folate plasma concentrations. Positive associations could be explained by an increased vitamin intake with a higher energy intake in the current study. However, this contradicts the observed negative associations. An explanation for these findings could be that the observed associations are very small and that any associations in the multivariable models are also influenced by the effects of other determinants in the multivariable models.

Although it is often assumed that physical activity leads to increased vitamin requirement, evidence for this hypothesis is lacking ^(^ ^39^ ^)^. Both in the univariable and in the multivariable models, we observed no association between physical activity and vitamin plasma concentrations. This observation is in agreement with studies in which no differences were observed in vitamin B12 and folate concentrations between groups with relatively low and relatively high physical activity ^(^ ^6^, ^7^ ^)^. However, in one of these studies, in which only women were included, a higher intake of vitamin B12 and folate in the high physical activity group was observed ^(^ ^7^ ^)^, which might explain the absence of differences in vitamin concentrations. In the women‐only study, it could be that differences in vitamin concentrations would have been observed between the two groups should vitamin intake have been similar in the two groups. However, in the other study in which no differences in vitamin concentrations were observed, folate intake in men and vitamin B12 intake in men and women were similar in groups with both low and high physical activity ^(^ ^6^ ^)^. In the present study, vitamin intake was also not different between different quartiles of physical activity; thus, a higher vitamin intake in subjects with a higher physical activity level cannot explain the lack of an association between physical activity and vitamin plasma concentrations.

The results of the present study suggest that sex, age and energy intake should be taken into account when studying the relationship between intake and plasma concentrations of vitamin B12 and folate. By contrast to our hypothesis, we did not find evidence that physical activity influences plasma concentrations of these vitamins. This suggests that it is not necessary to take into account physical activity level when studying the relationship between intake and plasma concentrations of vitamin B12 and folate.

In this physically active study population who did not use vitamin supplements, the intake of both vitamin B12 and folate equivalents can be regarded as adequate, considering that, for both vitamins, the median intake was higher than the EAR and only a small percentage of the population had an intake below the EAR. However, these findings must be viewed carefully because an FFQ is not the best method to evaluate adequacy of nutrient intake. Plasma concentrations of vitamin B12 and folate can also be regarded as generally sufficient, considering that more than 95% of the population had plasma concentrations within the reference range, and only 1.6% had deficient plasma concentrations. The results of the present study may therefore also suggest that people who are at least moderately physically active do not need a higher vitamin intake than persons with low physical activity to maintain sufficient plasma concentrations. In addition, it is likely unnecessary for physically active people to use vitamin supplements when their vitamin intake from foods meets the EAR. However, further intervention studies designed to specifically evaluate dietary requirements are necessary to experimentally confirm these suggestions.

To our knowledge, this is the first study in which the relationship between intake and plasma concentrations of vitamin B12 and folate has been investigated in a large study population, comprising both men and women over a broad age range, with varying levels of physical activity. A limitation of the study is related to the self‐reporting methods for the assessment of dietary intake and physical activity. Although we used validated questionnaires that are frequently used, all self‐reporting methods are prone to several types of error such as recall bias or the tendency to provide socially desirable answers ^(^ ^40^, ^41^ ^)^. A specific limitation of an FFQ is that single foods are grouped into groups of food items, where the variation of reported intake may be underestimated. This results in a smaller distribution of nutrient intake, and consequently an underestimation of the prevalence rate of inadequate intake. Therefore, reported prevalence rates of inadequate intake should be interpreted with caution. However, the median values and prevalence rates of inadequate intake observed in the present study are in agreement with values and prevalence rates observed in the Dutch National Food Consumption Survey ^(^ ^42^ ^)^ and a study among Dutch elite and sub‐elite athletes ^(^ ^43^ ^)^. In these studies, dietary intake was assessed with 24‐h dietary recalls, which have a higher precision in estimating the distribution of dietary intake ^(^ ^44^ ^)^. Also, evaluation of energy intake using the Goldberg cut‐off method indicated that under‐reporting was limited in the present study. Another limitation is that folate was analysed in plasma, which is known to be sensitive for transient changes in folate intake. Red blood cell folate is considered to be a more robust indicator of folate status than plasma folate because red blood cell folate reflects folate status over the last 3–4 months ^(^ ^2^, ^45^ ^)^. Synthetic folic acid has a higher bioavailability than natural food folate ^(^ ^28^ ^)^, which may therefore be a confounding factor in plasma folate analysis. It is worthwhile noting that fortification is limited in the Netherlands (maximum 100 µg of folic acid per 100 kcal) and that supplement users were excluded in the present study.

In conclusion, we observed a positive association between intake and plasma concentrations for both vitamin B12 and folate in people with a physically active lifestyle. By contrast to our hypothesis, physical activity was not a determinant of vitamin B12 and folate plasma concentrations. However, sex, age and energy intake were found to be determinants. Thus, when investigating the relationship between intake and plasma concentrations of vitamin B12 or folate, sex, age and energy intake should be taken into account.

## Transparency declaration

The lead author affirms that this manuscript is an honest, accurate and transparent account of the study being reported. The reporting of this work is compliant with STROBE guidelines. The lead author affirms that no important aspects of the study have been omitted and that any discrepancies from the study as planned have been explained.

**Conflict of interests, source of funding and authorship**

The authors declare that they have no conflicts of interest.

This study was financially supported by the EAT2MOVE project and a grant from the Province of Gelderland, proposal PS2014‐49.

AMB, MGJB and JMTKG designed the study. JdV was responsible for the FFQ. DSMtH and MTEH provided the data. AMB analysed the data. All authors interpreted the results. AMB wrote the paper. All authors critically reviewed and approved the final manuscript submitted for publication. All authors are responsible for the final content.
